# Social media analysis of car parking behavior using similarity based clustering

**DOI:** 10.1186/s40537-022-00627-x

**Published:** 2022-05-31

**Authors:** Nabil Arhab, Mourad Oussalah, Md Saroar Jahan

**Affiliations:** Faculty of ITEE, CMVS, University of Oulu, PO Box 4500, Oulu, Finland

**Keywords:** Natural language processing (NLP), Parking, Social media, Social networks, Twitter

## Abstract

This paper investigates car parking users’ behaviors from social media perspective using social network based analysis of online communities revealed by mining the associated hashtags in Twitter. We propose a new *interpretable* community detection approach for mapping user’s car parking behavior by combining Clique, K-core and Girvan–Newman community detection algorithms together with a content-based analysis that exploits polarity, relative frequency and dominant topics. Twitter API was used to collect relevant data by tracking popular car-parking hashtags. A social network graph is constructed using a similarity-based analysis. Finally, interpretable communities are inferred by monitoring the outcomes of clique, K-core and Girvan–Newman community detection algorithms. This interpretability is linked to the aggregation of keywords, hashtags and/or location attributes of the tweet messages as well as a visualization module that enables interaction with users. In parallel, a global trend analysis investigates parking types and Twitter influence with respect to both sentiment polarity and dominant trends (extracted using KeyBERT based approach) is performed. The implementation of this social media analytics has uncovered several aspects associated to car-parking behaviors. A comparison with some state-of-the-art community detection methods has also been carried out and revealed some similarities with our developed approach.

## Introduction

The increase of urban pollution along with the extensive use of cars and deficiency in public transportation services have transformed the transportation ecosystem [1], car traffic management [2] as well as the car parking planning and management services [3]. These transformations became a challenge for every municipality and cause frustration to individuals, business organizations and local authorities. On the other hand, the constant increase of urban population and the number of cars created a burden demand for car parking availability. This challenge affects both user’s communities and city urban planners. Understanding and examining the efficiency of car parking arrangements along with the eliciting user’s preferences and driving behavior is crucial and necessary in order to unfold potential parking issues and design appropriate strategy.

Loosely speaking, the presence and/or absence of car parking infrastructures in dense areas can affect city traffic, transportation ecosystem and emissions [4], which increases pollution [5], and can cause driver’s frustration. Moreover, location and availability of parking lots can have significant impact on the surrounded businesses ecosystem of the city [6]. For instance, the location of car parking infrastructure and its scale are found to affect the urban life in the vicinity area [7]. With the emergence of online platforms that enable user’s generated content with a single click, users’ encountered parking problems can be reported through online platforms and social media services such as online reviews, tweets, or posts. Analyzing the content of these posts can unfold various aspects of user’s car parking behavior and preferences such as parking time, length of stay, payment preference, car sharing potential, business incentives, opinion about public transportation system, among others. Similarly, reading consumer’s reviews about parking can influence user’s future choices, company’s planning and reputation as well as city planning [8].

In this context, social media grants a new class of communication models that allow people to express their thoughts freely about any subject/topic, to create and build communities or groups in an interactive and participator manner [9], which provides useful insights for community, policy-makers and researchers [10, 11]. The development of hashtag [constituted of a keyword or a phrase following the symbol (#)] based community construction, initiated by micro-bloggers to create a flow of information around a particular topic or trend, seeking contributions from other users, offers an appealing framework to discuss car-parking issues and users’ behaviors. This partly motivates our work in this paper, which aims to investigate the car parking ecosystem by analyzing the structure of online communities induced by appropriate hashtags in Twitter. The choice of Twitter is justified by its ease access data using various Twitter APIs as well as the fact that many professional organizations maintain active presence in Twitter together with the maturity of related data analytical tools [12, 13]. The collected dataset includes attributes like tweet messages, user ID, screen names and hashtags, which are then processed and adapted for applying social network and graph theory techniques in order to detect and identify relevant communities using an innovative *interpretable* social mining based strategy. The outcomes enable us to uncover hidden latent variables and parking issues that cannot be known straightforwardly to policy-makers and urban planners. Specifically, the motivation grounds for this work are at least threefold. First, as pointed out in [14], empirical knowledge about habitual behavior in the transportation literature is limited and mostly restricted to mode choice behavior and repetitive behavior in comprehensive activity-travel patterns, which calls on further research on the issue. Second, there are a variety of stakeholder groups that will benefit from this research. Indeed, any new knowledge in terms of drivers’ parking behavior would enable (i) policy planners to better monitor and refine policy accordingly; (ii) law enforcement officers to better identify likely scenarios of parking violation occurrence; (iii) city planners to better optimize existing resources; among others. For instance, it matters to know how the users are willing to park their car far from their destination, what factors that motivate users to accept multi-modal transportation, car-sharing or incentives, how user’s demographic attributes impact their parking choices and decisions. Third, the use of hashtag-based analysis enables us to further scrutinize the penetration of such social media data into transportation research. This can serve as a useful tool to generating traffic-relevant cues that help understand the root causes that prevent the public from and explore strategies for achieving the Target Zero goal [15]. The main contributions of the paper are fourfold.

- First, an approach for constructing a social network graph [16] from the hashtag dataset is constructed and analyzed in terms of characteristics of the underlined communities. The construction makes use of similarity score among tweet messages in the sense of Jaccard measure at token level and a threshold value inferred from the analysis of the giant component of the corresponding network graph.
- Second, an approach for revealing *interpretable* communities that makes use of distribution of common keywords, hashtags and location of users is revealed and implemented.
- Third, a global trend analysis that investigates both different parking types and engaging social media data is proposed. The analysis makes us of both polarity trend and discussion topics as revealed by KeyBERT-based method [17].
- Fourth, the application of the developed approach enables us to identify factors that affect user’s decision in terms of parking and elicit driver’s preferences in a way to help policy-makers design appropriate urban planning policies.

“Background” section of this paper describes the background of this research and some state-of-the-art approaches. “Data and method” section emphasizes data collection approach and the data analytics method. In “Results and discussion” section, the results are highlighted and discussed with respect to some related work in literature. Finally, a conclusion and perspective work are reported in “Conclusion” section.

## Background

Work in parking behavior has been promoted after the pioneering and seminar work of Polak and Axhausen [18], who, based on interviews with drivers, formulated eight tactical heuristics of parking search that drivers choose depending on available parking facilities, occupation rate, prices and expected dwelling time. The multiplication of transportation policies and urban development with the associated high demand for parking supply has opened the door wide for further research in the field. The sensitivity of parking behavior to pricing has been subject to several studies to comprehend the association between parking cost and user’s behavior [19, 20] who considered the aggregate consequences of changes in parking prices. Lehner and Peer [21] summarized the results of more than 50 studies regarding the price elasticity of three factors—parking occupancy, parking dwell time and parking volume. The authors showed that drivers are more sensitive to parking prices when alternative transportation modes are available, whereas daily commuters are the least sensitive among driver groups to parking prices. On the other hand, with the availability of large scale dataset, gathered through questionnaire, mobile apps, social media or smart parking systems,^1^ research on applications of data analytics, machine learning or statistical inferences to parking behavior analysis has seen a renewal interest. Some of these interesting and related studies are summarized below.

Mondschein et al. [22] evaluated users’ sentiment that characterizes existing car parking supply as reflected by the online reviews related to parking collected from Yelp restaurants reviews in Phoenix, Arizona regions. In their study, the sentiment analysis revealed that a negative emotion is often associated with parking reviews. In addition, this has shown to affect many businesses in the region where low rating scores have been recorded in the vicinity of car parking locations. Zhang et al. [23] adapted the Bayesian network approach to analyze the individuals’ parking behavior by standing on the multi-information. They focused on investigating the impact of some parking factors that influence the parking search decision, such as parking fees, discounts, and drivers’ preferences when choosing a parking space. They found that younger vehicle owners and women are more likely to select parking lots with a parking fee discount. Spiliopoulou et al. [24] analyzed the parking behavior from the legacy perspective using a staged dataset, which allowed them to perform multiple timely comparisons in order to identify the factors that cause and increase/decrease the illegal parking phenomena in Greece. The study revealed the tendency of people to park as near as possible to destination regardless of legal or illegal parking spaces, encouraged by inadequate lot capacity and low enforcement level. Likewise, Aljoufie [25] investigated illegal parking topic and its behavior in the Jeddah region to identify sites and periods of the days where illegal parking cases occur. In the same spirit, Meng et al. [26] considered Wuhu region in China as a case study to investigate the parking behavior and its characteristics through a set of field observations about parking spots utilization. Their findings revealed issues of high cost in space renting and a lousy parking management system. The study has also proposed some protocols and solutions to overcome the already detected problems, such as optimizing parking layouts and smart car parking management systems. Zong et al. [27] investigated drivers’ preferences in choosing specific parking lots and the impact of fee discounts in Beijing area using a Bayesian network based approach. Especially, they applied structural equation modeling method to reveal the impact of some parking attributes such as family influence and parking fees on the parking decision. Their results showed the importance of family ties and preferences on parking choices. They also showed a direct correlation between the parking cost and parking duration. Feng et al. [28] studied the possibility of predicting parking behavior in Ningbo, Zhejiang city of China using 396-day parking data from shopping mall. They showed that random forest classifier achieves best parking behavior prediction accuracy of 89%. In [29], the authors focused on the on-street parking in Rajkot city in India aiming at identifying parking rates between various land-use patterns using some empirical analysis based approach. The data were collected using license plate inventory at different time intervals. In [30], the authors investigated parking occupancy with respect to user’s choice and preference using a questionnaire like analysis that involves a number of social and demographic patterns (e.g., parking price, trip purpose, on-street versus off-street). Using a linear regression-based method, the authors provided an estimate of the parking demands and related parking characteristics that impacts the drivers decisions such as the distance from parking place to the destination, parking lot availability, among others. Chen et al. [31] analysed the choice behaviour of people for surface parking lot using fuzzy multiple attribute decision making process for optimal parking space choice. Ni and Sun [32] advocated agent-based modelling approach to assess the impact of parking reservation system (PRS) on parking behaviour. Gaming theory has also been applied to uncover some insights regarding parking behavior. In this context, Bonsall and Palmer [33] developed traffic simulator to estimate drivers’ reactions to parking prices and off-street parking facilities. While, Ben-Elia and Avineri [34] proposed the PARKGAME serious game platform to gain in-depth insight into driver parking behavior. The preceding demonstrates the usefulness of data mining technologies, including machine learning, social media analytics and gaming to understand public opinion associated to parking behavior and transportation research. Table 1 summarizes some of existing work in the field of big-data car parking analytic are summarized in Table 1.

**Table 1:** Literature table

Authors	Data used	Method	Results	Limitations
Zhang et al. [38]	Data collected using surveys, and questionnaire (3000 questionnaire delivered, and 2586 validated)	Use K2 algorithm with maximum-likelihood function to estimate parameter learning for Bayesian network. The nodes of the network holds the various factors that influence the parking decision and the edges reflect relationships	Identification of the age and gender as factors influencing selection of parking spot. Women and young people tend to select a spot with discount more than others. This provides suggestions to business owners and discount distribution for parking	The predictive model for parking fee needs more research and optimizations in order to improve its accuracy
Zong and Wang [39]	Parking data collected from the area of Beijing, China. Based on large scale surveys 46,874 parking records obtained	Investigate parking behavior, and parking search decisions by utilizing K2 algorithm, and Bayesian learning techniques to develop the Bayesian network	The parking duration, and parking location are influenced by parking period time	Limited number of parking aspects were used in the analysis
Spiliopouloua and Antoniou [24]	Traffic data gathered from previous studies from 6 different regions in Greece	Comparative study, and analysis of the parking diagrams in a staged approach	Identify illegal parking behavior and parking preferences were elicited illegal parking occurs when people don’t find free legal spaces, together with the absence of authority systems	The study was limited to only some regions from Greece
Teknomo and Hokao [40]	Data collected by performing questionnaires with: Parking users about their parking behavior in terms of travel and individual infos and their preferences Government officials about parking strategies programs	Analyze data using various model-based location such as regression, analytic, hierarchy to identify influence of parking, behavior especially in business areas	Revealed some factors that influence search decisions in business areas, e.g., location, duration of parking search	Parking models need more development in order to impact parking policies
Wang et al. [48]	Collect illegal parking reports from users via a mobile app	Mobile app system integrated with Wechat app as a social media platform used to report illegal parking	Contribute security by integrating the mobile app with police systems to minimize illegal parking in the city	Limitation with the accessibility of such system to contributors The system was only integrated with one mobile App
Mondschein et al. [41]	Parking data collected from online reviews of a business, Yelp restaurants in the region of Phoenix, Arizona, US	Analyze the sentiment that accompanies the parking online reviews	Negative sentiment often accompanies the parking online reviews Reviews where parking was mentioned give less rating	The results are moderate, and small effect is noticed in terms of the impact about businesses and ratings
van der Waerden et al. [42]	Data collected using online questionnaires related to business trips, e.g., vehicle use, time, area. Questionnaires distributed to residents in cities of Hasselt and Genk in Belgium. 436 responses	Statistical analysis of the questionnaire data, such as frequencies, percentages, and multinomial regression analysis	Parking behavior analysis in business areas. They revealed that individuals tend to use their cars in business areas, and in a regular way	Only some parking issues and aspects are treated. Limited to analyse the parking behavior in business area. The size of the data used in the study is small and does nor reflect official national statistics

Apart from car-parking domain, the application of social network analysis to uncover user behavior and patterns. For instance, Kanavos et al. [35] explored the relationship between user behavior and their emotions using Twitter data and social network analysis. Their method evaluate the influence of user actions and behaviors by modeling and identifying communities based on the level of influence. Similarly, Li et al. [36] applied social network principles and hierarchical clustering to identify various communities associated with distinct facets of user behaviors. Their approach uses the followers and following relationships to create social network graph and then track personnel tags posted by the users. Opinion community and opinion leader detection are explored in [37]. In the opinion community leader model, a social network is constructed to map users’ thoughts and interactions with opinion community. Various competing models were tested in a cloud environment where the results demonstrate the performance of opinion detection communities.

## Data and method

### Data collection and preparation

The dataset used in this study is collected using Twitter Streaming API. The GetOldTweets3^2^ python library is used for data scraping. Three leading car parking related hashtags were used in the queries made to Twitter API: #parking, #parkinggarage, and #parkingspot. The choice of these hashtags is motivated by their high exposure rate (as quantified by https://best-hashtags.com/hashtag/exposure/) and relevance in terms of their car parking content. Next, multiple attributes were collected for approximately four months, starting from 1st January 2020 until 11th April 2020. The dataset includes the user’s Identifier (ID), the screen name, the tweet text, the hashtags, the location (if available), and the time of the tweet. In overall, the dataset contains 10551 tweets related to parking. It should be noted that although specific hashtags were used for Twitter data, it often occurs that the collected Twitter data include mentioning to other hashtags as well, which explains the large-scale dataset of hashtags collected as well.

In order to utilize the collected Twitter dataset and explore its content, an initial preprocessing stage is necessary in order to filter out noisy terms and normalize the content in a way to maximize the outcomes of standard NLP modules. This task follows the standard text mining approach, which starts by converting to lowercase characters all tweet text, screen names and hashtags, then a tokenization task was used to distinguish various tokens in tweet text message. Next, noisy terms including stop-words^3^ were removed, together with punctuation and non-desired characters. This process excludes some important characters (like @) and User-IDs as this is required to distinguish retweets and Tweet identities.

### Parking global trend analysis

In this part of the approach, the aim is to explore the parking trends and preferences of the users and deliver a global view of the users’ demands, likes, and dislikes regarding parking search decisions. For this purpose, two techniques have been utilized. The first one makes use of sentiment analysis using SentimentVader [43] capitalizing on the valuable insights that can be inferred from tweet message content in terms of positive and negative polarity. The second method explores the content of tweet messages in terms of generic trends and topical description. For this purpose, we used the deep-learning architecture provided by KeyBERT [17], which uses the pretrained model of BERT for a keyword extraction from textual sources. In essence, KeyBERT creates N-gram elements, then uses cosine similarity to measure the similarities between each candidate answer and the tweets document, so that only highly scored candidates are preserved.

Furthermore, we considered two-level of analysis:

1. *Parking type-based analysis*. In this case, we shall gather all data associated with an individual parking type and perform both Sentiment-based analysis and KeyBERT-based analysis. The former allows us to extract user’s feelings and opinion about the given parking type. While, the application of KeyBERT expects to shed light on vital sentences and keywords that could point out some users’ demands, likes, or dislikes. The considered parking types are On-street, Off-street, Underground, and Airport parking. This choice is motivated by the dominance of these parking types in literature as well as by an initial exploration stage of our dataset.
2. *Engagement-based analysis*. In this case, we shall consider only those tweets that convey high engagement from users, and then apply the sentiment and KeyBERT to unfold the polarity and topical content of a such data. This aims to identify important factors that influence parking decisions from discussions that convey high level of users’ interactions and engagements. For this purpose, in the same spirit as [44], we shall will assume that a given tweet conveys high engagement if it is either retweeted or liked by at least one user.

**Fig. 1:**
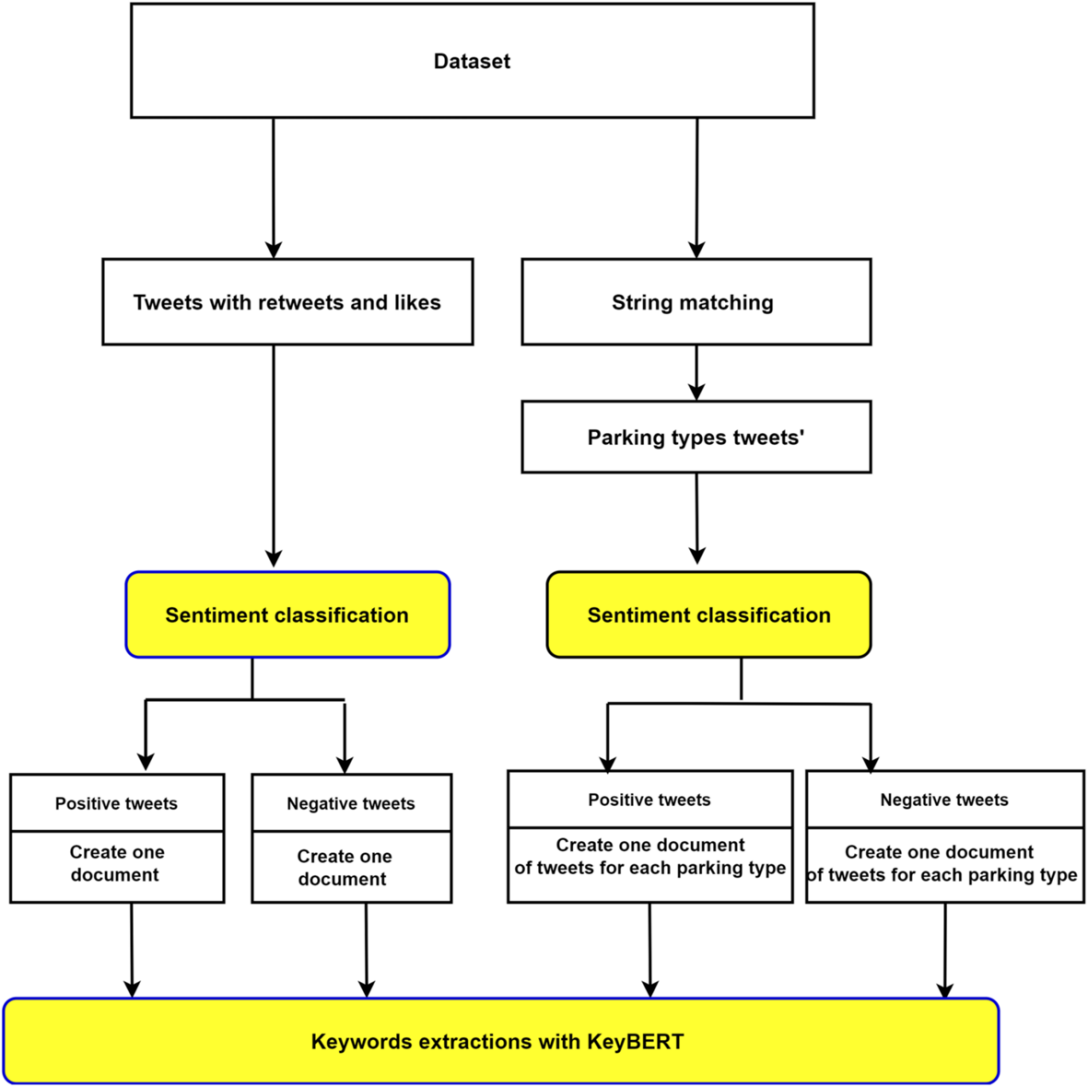
High level diagram of the parking global trends analysis

Figure 1 provides a high level diagram description of this global trend analysis.

### Construction of the similarity network

The essence of the method pursued in this paper consists in building a social network graph from the collected Twitter dataset. In this respect, the nodes of the network correspond to user’s IDs while a link between nodes, say, $ID{_1}$ and $ID{_2}$, is established whenever there exists at least one tweet message (excluding retweet) generated by $ID_{1}$ that is found sufficiently similar to that of $ID_{2}$. This textual similarity is quantified using standard Jaccard similarity [45] score,^4^ which computes the amount of overlapping among the two texts. In other words, an edge between $ID{_1}$ and $ID{_2}$ is established if and only if, there exist (non-retweet) Twitter messages $T_{1}$ from $ID_{1}$ and $T_{2}$ from $ID_{2}$ such that:

$$J(ID_1,ID_2) = {\frac{|T_1 \cap T_2|}{|T_1 \cup T_2|} }\ge \gamma$$

where $\gamma$ stands for the Jaccard similarity threshold beyond which the assertion “the two Twitter IDs ($ID_1$ and $ID_2$) are deemed to share sufficient textual content” is valid. See also Algorithm 1 for a detailed algorithmic description. Strictly speaking, as part of the dataset domain structure, since each tweet message, after the preprocessing stage, is represented by a list of tokens/words, the calculus of Jaccard similarity score turns out to a simple count on the total number of common words/tokens among the two texts $T_{1}$ and $T_{2}$ over the total number of distinct tokens among $T_{1}$ and $T_{2}$. This yields a similarity score ranging in the unit interval where zero would indicate no overlapping token, while a value one corresponds to a fully matching content in terms of tokens. Although this does not necessarily entail similar tweet messages due to potential impact of preprocessing stage and the negligence of token ordering information. On the other hand, the choice of the threshold $\gamma$ should be very much dependent on the prior knowledge about the frequency and the nature of textual communications held by $ID_1$ and $ID_2$. Therefore, a cautious and a contextual analysis should be followed to select appropriate threshold to ensure a rational graph construction. This selection process is performed by monitoring the size of the giant component (see next subsection) to ensure a critical network size is reached. We shall mention that alternatives to Jaccard similarity measure are also studied elsewhere. Gali et al. [46] provided an extensive comparison of potential similarity measures at character, token, n-gram and semantic level together with their associated implementation toolkits. Their findings highlights the importance of nature of dataset as the key factors that guides the selection of appropriate measure.
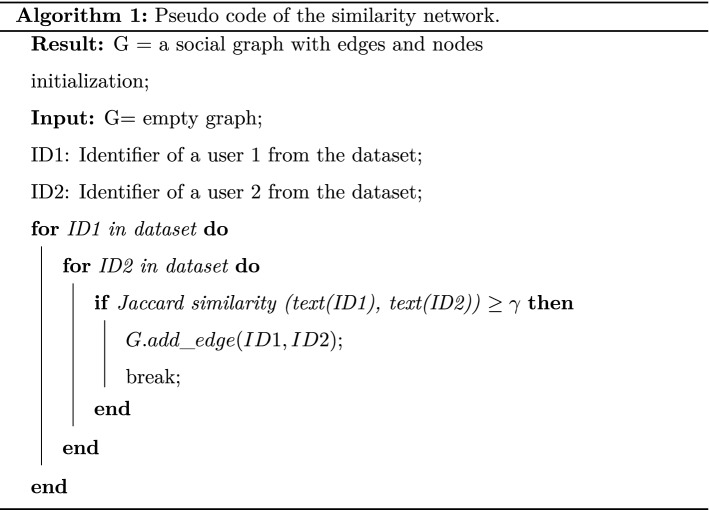


#### Community detection algorithms

In order to analyse the constructed social graph and provide interesting interpretations of the results, common community detection algorithms whose implementations are available in popular machine learning libraries were explored, and then we restricted to three algorithms who exhibited good satisfaction at the exploration phase: Clique, K-core and Girvan–Newman, briefly described below.

Clique is a type of community, which corresponds to a sub-graph where each node is connected to every other node of this sub-graph. Namely, a clique of size m is such that each of its nodes has a degree equal to m − 1 [47]. This corresponds to a high constraint in the community construction.

K-core is a less restriction than clique-like community and corresponds to a maximal connected subgraph in which each vertex has a degree at least equal to k. The higher the value of k, the higher the tendency of the underlined community towards a clique. The construction method for k-core identification is based on repeating deletion process of all nodes with less than k vertices connected to them [48].

Girvan Newman: is one of the most popular construction algorithm for online community detection. It is based on measuring edge betweenness values in the graph and involves several runs. The first step determines the edge betweenness value for each edge of the graph. Second, one selects the highest edge betweenness value, and deletes all edges (and nodes) that are associated with it. Third, one calculates the edge betweenness scores again for the remaining edges in the graph. This process is repeated for phases 2 and 3 until no edge remains [49].

### Interpretation of communities generated by the similarity graph

**Fig. 2:**
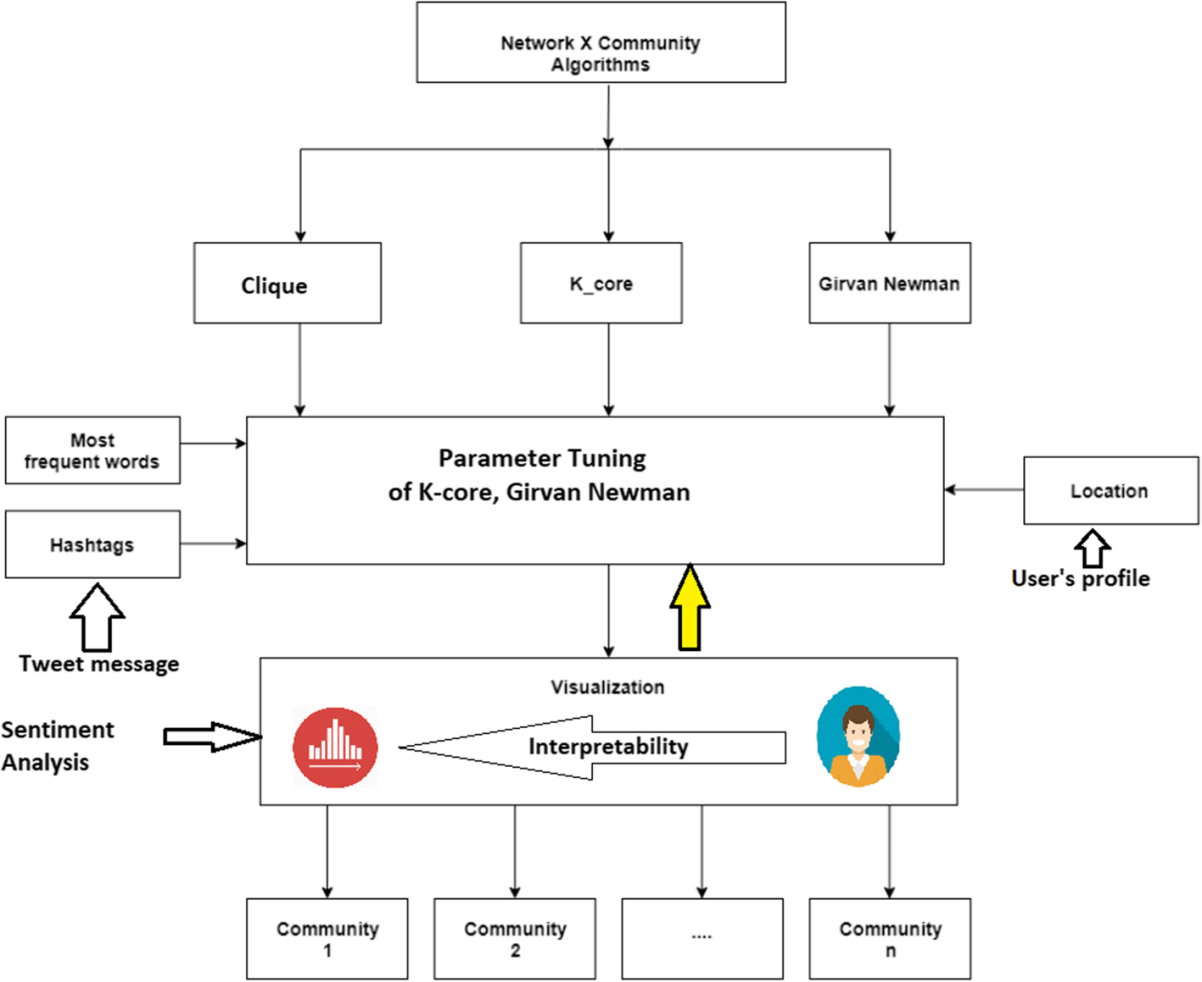
High level diagram of the community interpretations

The communities induced by the application of clique, k-core, and Girvan Newman algorithms were analyzed, visualized and interpreted. The core idea in this matter is to restrict the community detection to only those that can easily be interpretable according to three specific aspects: frequent keywords/topics, list of hashtags and location information as inferred by user profiles. This process follows a semi-automated reasoning where the communities generated by (k-core, for different choice of k), Clique and various levels of Girvan–Newman algorithm are scrutinized by monitoring the most common keywords, hashtags and locations and see whether they can be assigned to a common umbrella, and therefore validate the underlined community. For this purpose, the tweet messages pertaining to the same identified community are compiled together, and histograms of the ten most frequent keywords, hashtags and locations are constructed. Then, a human annotator scrutinizes these histograms to find out whether a common characteristic in terms of either a prominent (sub) topic (from either frequent keywords or hashtag list) can be recognized; or whether the users of the same community belong to the same location. The identified communities using the above process are visualized using appropriate visualization tools provided in Python library NetworkX.^5^ Especially, this analysis was performed for multiple subgraphs generated by Clique, k-core and Girvan–Newman algorithms. This semi-automated process for generating interpretable (sub) communities is illustrated in Fig. 2.

## Results and discussion

In this section, the experimental results of the above analysis are presented. We distinguish global trend based analysis results and community-based analysis results.

### Global trend analysis

#### Parking type results

In this part, we present statistics and findings related to users’ preferences and concerns regarding each parking type. Table 2 presents, for each parking type, the statics in number of supporting tweets, sentiment polarity and the main issues raised by the users as identified by KeyBERT-based analysis. Besides, we separated the KeyBERT results for positive polarity and negative polarity cases to comprehend the concerns of each user category. As shown in Table 2, airport parking is by far the most popular parking type (81.6%), which was identified using string approach like method. Besides, looking at the opinion of the users who discussed airport parking, revealed that the majority 63% have rather positive opinion, compared to 11% negative opinion. In terms of issues raised by the users, it turns out the discussions focused on the need of airport parking, its ease access with multiple modalities and availability. Besides, the negative polarity users were mainly concerned by the limited services offered at parking facility and the possibility of ticket refunds and sudden price raise. This finding overlaps to a large extent with the research outcomes of [50] about airport parking where parking providers needed to build more parking lots even at small airports. This research also highlighted the parking operating revenues that can cover to some extent the increasing safety and maintenance cost of such infrastructures.

**Table 2:** Parking types and corresponding statistics

Parking type	Nb T	PT (%)	Neg T (%)	Neut T (%)	keyBERT element	
					Positive KeyBERT elements	Negative KeyBERT elements
On-street	40	50	20	30	Onstreet parking guidance Onstreet parking free Onstreet parking great Onstreet parking bylaws Parking require continued	Pay park onstreet New onstreet charges Onstreet charges rise Park onstreet auspicious Park onstreet auspicious
Off-street	11	91	9	0	Free parking saturday Free parkingparking activities Parking saturday Commuter parking activities	Spaces city required Offstreet spaces city Spaces city required Publicly mandated offstreet spaces
Underground	34	47	14	38	Beautiful apt rera permit Entrance underground facility Accommodation parking garage Underground selfparking offer	Fees city edmonton Parking fees Edmonton curbside epark Parking facilities
Airport	377	63.3	11.67	25	Parking best airport Airport parking options Need airport parking Airport parking safe Make airport parking	Refund airport parking Airport parking services Airport parking limited Priced airport parking Parking scam

*Nb T* Number of Tweets, *PT* positive tweets, *Neg T* negative tweets, *Neut T* neutral tweets

The second parking type in terms of popularity is the on-street parking (8.6%), shows the majority of users (50%) have positive opinion compared to 20% negative opinions. While the discussed topics turn around enforcement, regulations, increase in parking supply and closeness with respect to city centre. Besides, there were demands and critics concerning to the applied fines. The popularity of this parking type is explained by its efficiency as it often enables users to reach their destinations more quickly than indoor or off-street parking type, despite the critics on fine regulation and sudden price raise. This finding is in agreement with work reported in [51], which, after reviewing the state-of-the-art of on-street parking, concluded that a such parking category should only be provided in minor/secondary roads and avoid main roads to enhance pedestrian security. In parallel, the report also highlights the need to provide alternative parking supply to offset the pressure on limited on-street parking availability. The third parking type is underground parking (7.4%). Similarly to airport and on-street parking, the majority of users have positive opinion (47%). Their discussions revolved around the pricing, ease of access location, underground available facilities and the quality of the environment in the vicinity area. Moreover, in this type of parking, multiple tweets were more business-centred by offering special fairs, reduced parking prices, and advertisement to new infrastructures. Finally, the less discussed parking type—off-street parking—is characterized by the overwhelming dominance of positive sentiment reflecting the users’ overall contentment and satisfaction. The discussed topics are centred around free parking availability, especially on weekends as well closeness to city centre and the availability of activities in the vicinity. Some of these outcomes have been discussed in other related work. For instance, in [52], the authors showed that the parking cost has the dominant influence on users’ parking decisions.

#### Engagement-based analysis

Table 3 shows the results of the most engaging tweets according to like/retweet assessment as well as the corresponding users’ reflections, parking demands, critics, likes, or dislikes. In total 3486 tweets were found to belong to this category, where each tweet has at least one interaction, either through like or retweet. Looking to Table 3 reveals that the positive sentiment is pretty dominant (55% of the tweets), and only 15% were negative. The KeyBERT analysis exposed some of the key sentences that characterize the parking from users’ point of view. We distinguished the views of users with positive opinion and those with negative opinion as well as the view of overall users regardless their polarity. For instance, the overall population analysis shows an interest to availability of parking in commercial areas, residential areas, on-street and nearby their destinations. Mapping positive polarity tweets revealed the users’ interest to share good locations and free parking availability. Interestingly, in negative polarity case, we notice the tendency of users to propose solutions in areas such as residential parking and referred to it as a crisis. Other push towards industry-based solutions and review of regulatory framework and new management schemes. These user’s based reflections found from analyzing these tweets can be categorized into three main categories as follow:

- Need for more parking infrastructure, particularly in businesses or downtown areas.
- Lack of parking supply in both residential and business areas, which caused stress and frustrations.
- Poor management and regularization of land and parking lots.

**Table 3:** Analysis of tweets with likes and favorites

Nbr of tweets	3486
Positive tweets (%)	55
Negative tweets (%)	15
Neutral tweets (%)	30
KeyBert for all tweets in the list	Solution car parks; local car parks; market car park; completion car park; road car park; commercial areas parking
KeyBert for positive tweets	Queensland finfeed parking; living parking downtown; meet parking needs; parking town free; solutions urban parking
KeyBert for negative tweets	Solution car parks; residential parking crisis; parking solutions news; park residential streets; parking curbside instead; parking spaces wish; parking industry solutions; stress finding parking; unregulated car park; manage car parks

### Similarity graph construction

**Fig. 3:**
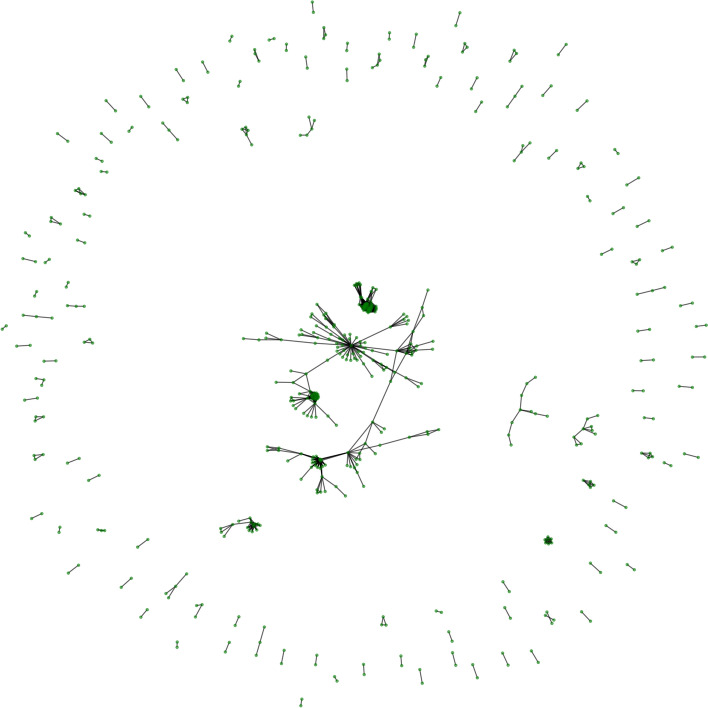
Similarity network

Following the methodology highlighted in “Parking global trend analysis” section, using a fine-grained tuning of Jaccard similarity threshold by varying the parameter $\gamma$ from 0.1 till 0.9 at incremental step 0.1 and monitoring the size of the giant-component of the induced graph, it turns out that setting $\gamma = 0.4$ yields the largest giant component before its starts to shrink drastically (smaller values of the threshold yield unattractive scenarios where most of nodes were connected). We therefore adopt this choice in subsequent analysis. Figure 3 displays this generated network-based similarity, while Table 4 summarizes the main attributes of this graph, which include network’s size, average path length, average degree centrality, clustering coefficient, path length. One notices that the graph has a modest number of connections between different nodes and sub-graphs, which show that the graph is somehow stretched and not tied. The giant component’s size represents 31% of the graph’s size, which will be further decomposed into various subcommunities. In overall, the average degree centrality and betweenness centrality values are rather low, which reflects the low connectivity between the graph nodes. In contrast, the relatively high average clustering coefficient value suggests some potential for extra (sub) communities and clusters.

**Table 4:** Summary of similarity graph

Attribute	Value
Number of nodes	533
Number of edges	1211
Size of giant component	168 nodes, 412 edges
Average path length	5.7084
Average degree centrality	0.0085
Clustering coefficient	0.3582
Diameter	14
Average betweenness centrality	0.0009

#### Computational complexity

Regarding the time complexity of the method and construction of the network, it should be noted that the data processing time was pretty reasonable for graph construction methods. Indeed, tested on a laptop HP with Processor Intel I5 5300U CPU 2.30 GHz, it takes 230 s to iterate through the different steps and complete the data processing. However, the graph construction takes exactly 16   min and 15 s to choose the nodes and build the connections. This time complexity could be significantly improved by adopting a multi-thread architecture and using GPU like machine (Table 5).

**Table 5:** The time complexity for data processing and the exact execution time for 1 MB data

Machine	Laptop HP with Processor Intel I5 5300U CPU @2.30 GHz
Operating system	Windows 10
Data processing time	230 s
Execution time (graph construction)	16 min and 15 s

### Interpretable community based analysis

Following the reasoning highlighted in “Construction of the similarity network” section, we apply the three community detection algorithms (clique, k-core for various values of k, Girvan–Newman), and monitor the interpretability in terms of most common keywords/hashtags and location of the Twitter IDs. For instance, using a simple frequency based analysis of the keywords constituting the tweet messages involved in one of the identified (sub) community generated by Girvan–Newman algorithm revealed the two most frequent keywords be “parking”, “free”. These can be cast into the generic topic of “free parking”. Similarly, in another community, the most two frequent words are “bad” and “parking”, which can be cast into a “bad parking” like community. This process enables us to discover communities related to airport parking, public parking, parking spot repainting, street parking, city parking, parking cost and fine, parking maintenance, traffic and corona-virus, among others. The choice of our topics is also motivated by the desire to elicit users’ parking preferences and concerns. The first central aspect is related to the factors that influence parking search decision, such as free parking, cost of parking, and parking spaces, which reflect an interest in knowing the availability of spaces. The traffic and transportation is another significant aspect, reflected by airport parking, public parking, street parking, city parking. Surprisingly, the impact of artistic flavor is also noticed through topics like “street arts”, “painting” and “street photography”.

Regarding location scrutinizing, we noticed for instance that common locations of the users as revealed by the users profiles were: India, The United States, Canada and The United Kingdom. Nevertheless, the analysis did not show up a clear path towards location-based (sub) community. Below are described distinguished communities identified using the process described in “Construction of the similarity network” section where four distinct communities were distinguished. Especially, the analysis of the identified communities showed that the communities acquired with clique and k core algorithms were associated with almost the same topics. Also, many of the (sub) communities were so small (i.e. just from 2 to 4 users) that analyzing them alone did not seem meaningful. That is why we restricted to the most representative ones only that we present next.

#### k-Core community

**Fig. 4:**
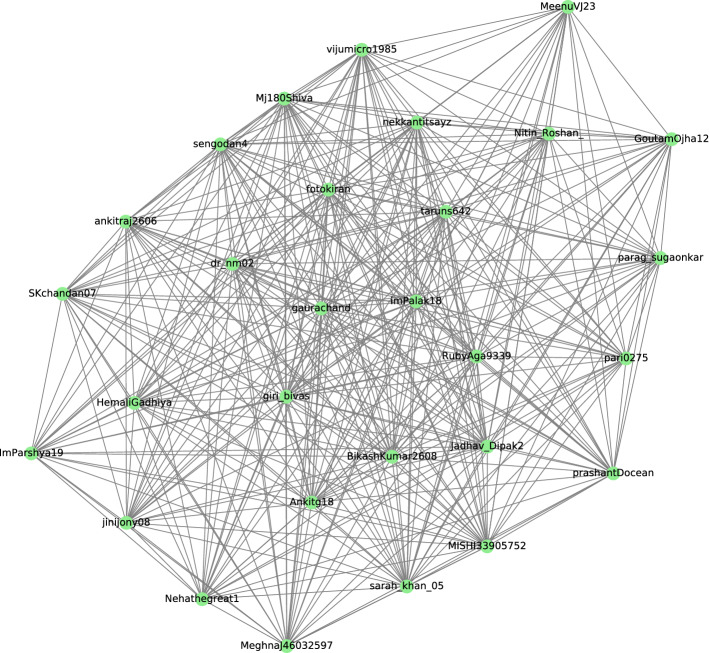
21 core community

This community is illustrated in Fig. 4. The community is a 21 core visualization. It shows a group of people participating in a competition hosted by a parking provider company that provides automated parking solutions with an app that helps vehicles move in and out of the buildings. It also provides free parking spots. Table 6 summarizes the most frequent words and the hashtags retrieved from the tweets which form this community. Based on this, the topic of discussion was mainly about using the mobile app for parking reservations, and inviting friends to participate in the competition hosted by the company.

The participant’ locations are all from India, but multiple cities have participated, 16 different locations were mentioned. The intensive positive social interaction around the community in terms of the topic of discussion, the number nodes or distinct participants and community size is extremely high, which symbolize the critical interest of the individuals in using intelligent solutions such as mobile applications either to quickly find parking lots in congested areas or to make reservations and communicate efficiently. Also, the community size and their interest overlap with the outcomes of the research work conducted by Siuhi et al. [53] and their results about the necessity of a mobile application for parking and the significant potential and impacts either on individuals parking search decisions or the environmental impact by reducing the car emissions and traffic congestion.

**Table 6:** Hashtags, and most frequent words within the 21 core community

Most frequent words	Hashtags
Friends, join, car, 6, ans, tagging, total	#parkwheels, #parking, #challenge, #contestalert, #puzzle, #findthecar, #contest

#### Pavement parking community

**Fig. 5:**
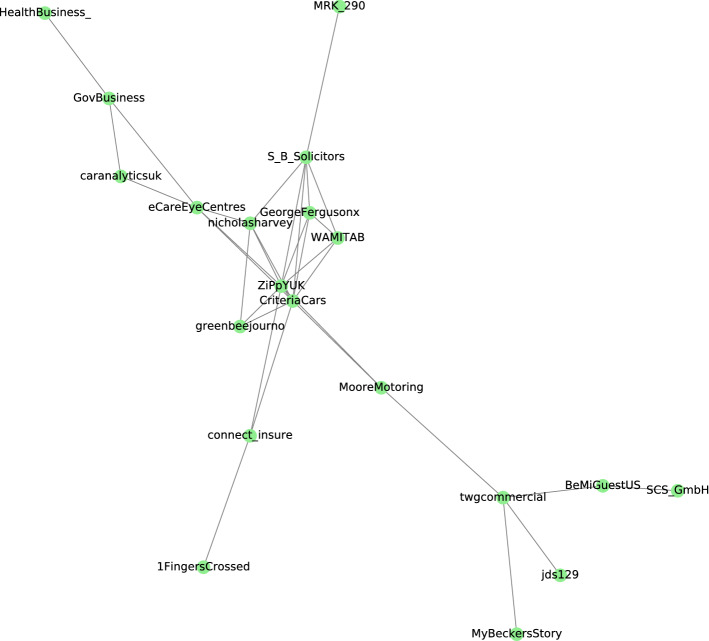
A community found from level 10 of Girvan Newman algorithm. Topic of discussion was banning of pavement parking in England

Figure 5 presents a community from level 10 of Girvan–Newman in which people were talking about banning pavement parking in England during the coronavirus pandemic in around 17 different cities and locations. Table 7 presents the most frequent words and the hashtags within this community. These results reflect the negative impact of the pavement parking and the coronavirus pandemic on England’s traffic and parking infrastructures. It indicates the pavement parking has provoked and caused residents’ frustration. The users tweeted about potential solutions to this effect. Usually, pavement parking creates problems for pedestrians and vulnerable groups such as people with limited mobility, disabled people, individuals with limitations in visibility. Moreover, it affects the pavement length by reducing the space for pedestrians. This joins the increasing research findings about parking violation [48] and illegal parking [24], which are found to be among significant issues and factors affecting the parking search decision of individuals and traffic congestion in cities. For instance, Wang et al. [48] built a datatset by collecting the daily police department reports in one of the China cities, and concluded that 35% of the registered claims in the city were about the parking problems including pavement parking. The authors also proposed a solution using a mobile application and an online platform for reporting illegal parking in the city.

**Table 7:** Hashtags, and most frequent words within the pavement parking community

Most frequent words	Hashtags
Banned, pavement, pedestrians, pandemic, rule, pose, England, panel	#coronavirus, #parkingnews, #pavement, #pedestrians, #accessibleparking, #parkingfail, #driving, #avementparking

#### Marketing community

**Fig. 6:**
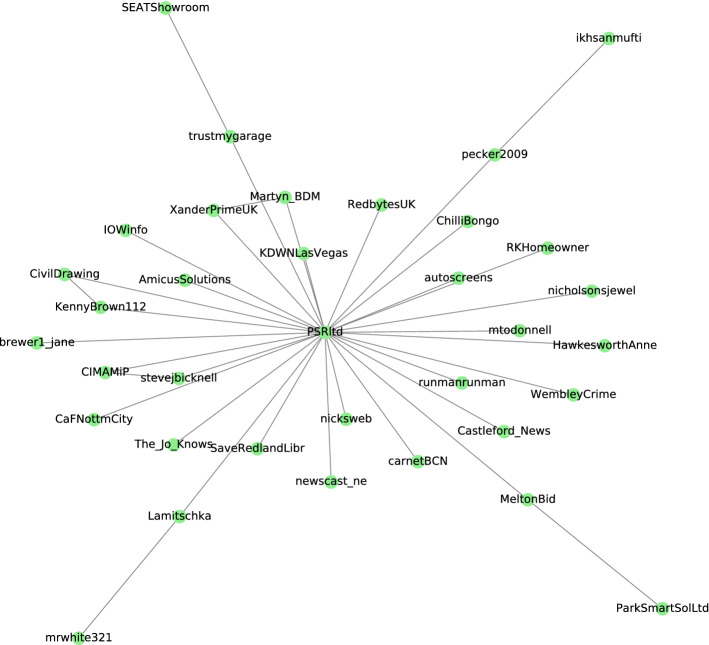
Community found from level 10 of Girvan Newman algorithm

The community presented in Fig. 6 was identified using Girvan–Newman algorithm. The social interactions and the type of influencers in this community expose the marketing and commercial aspect of parking in social media networking. According to the most frequent words, and the hashtags occurred within the community shown in Table 8, the topic of discussion sounds associated to parking near hospital premises during corona time, where the pandemic situation has caused problems with parking pot availability. Some people discussed the need to free parking spaces near hospitals. Others were trying to report the illegal parking caused by some individuals utilizing the parking reserved for disabled people. The community’s central node is a company and a big influencer called PSRltd,^6^ a marketing specialist and a parking provider in the United Kingdom. The company played a role in the pandemic by providing free parking for hospital workers. They were motivating and calling people to contribute by facilitating the parking for the care workers to park easily. Moreover, they have provided a website specialized in helping the care workers to reserve parking easily.

**Table 8:** Hashtags, and most frequent words within the marketing community

Most frequent words	Hashtags
Private, spaces, charges, unfair, fight, fines, invoices, staff, permits, residents	#hospital, #scams, #bluebadge, #disabled #covid19, #newport, #station

#### Event community

**Fig. 7:**
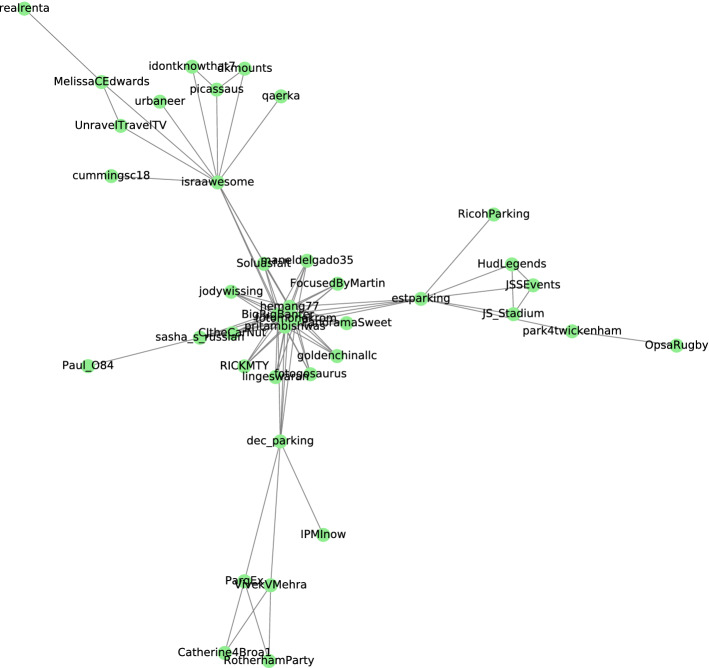
Community found from level 10 of Girvan Newman algorithm

The last community to be presented from the similarity network is shown in Fig. 7, along with its most frequent words and hashtags tabulated in Table 9. This community is related to social events and traveling. People in this community talked about the booking and pre-booking of parking spaces before going to the event. This interest indicated the individuals’ behavior regarding parking reservations in an event or travel. The pre-booking is the tendency by utilizing a mobile app for parking reservations and proceeding with an online payment rather than traditional parking approaches and on-site payments. This community shows an interest in using the new IoT and web application features such as E-payments, E-parking, automated *parking systems*, and parking reservation systems. All these factors and characteristics were identified by Revathi et al. [54], and Lin et al. [55] as essential factors that influence the individual’s parking reservations and decisions.

**Table 9:** Hashtags, and most frequent words within the event community

Most frequent words	Hashtags
Saturday, prebook, attending, concert, june, game, heading, forward, seeing, disappointment	#concert, #greenday, #mobility #hobby, #filmphotography, #light, #parking

### Alternative community detection algorithms

In this section, we have tested alternative state-of-the-art algorithms for community detection to compare the results with our approach that used only Clique, k-core and Girvan–Newman. We restricted our implementation to those algorithms whose software is publicly available. About ten different algorithms were therefore considered, although some returned void communities or failed to deliver desired patterns. Among algorithms tested positively one shall mention principled_clustering [56], belief [57], Fluid Communities [58], leiden [59] and chinesewhispers [60]. Other tested algorithms that failed to deliver interesting outcomes include Diffusion Entropy Reducer graph clustering algorithm (Der) [61], gemsec [62], walktrap [63], cpm [64], kcut [65], edmot [66] and lswl_plus [67, 68].

The outcomes of each algorithm from the above list is as fellow:

- Der, returned only two communities with very diverse discussions and locations. which render the interpretation of the results rather difficult, if not impossible.
- Gemsec, returned an error.
- Walktrap, returned 13 communities where three were fond similar to our findings (marketing, event, and pavement communities), while the remainder were very redundant and difficult to ascertain any interpretation. However, these results were not added to the discussion table.
- Cpm, returned an error.
- Kcut returned 6 communities; 5 of them contain a maximum of 2 nodes only, and one giant community. The results were almost ad hoc from interpretation perspective.
- Edmot returned error.
- Lswl_plus, returned error.

We shall mention the prospects of recent work by Sieranoja et al. [69] using k-means algorithm for graph clustering where high performance results were reported. This provides insights to our future in the field.

Table 10 presents the results of those algorithms that delivered non-void communities. We presented also in the table the overlapping with our community-detection algorithm in terms of detected communities as well as the characteristic in terms of size, main hashtags and top frequent words in the associated tweet documents, of any additional community detected. It is easy to see that all the algorithms bear some similarity with our (k-core, clique, and Girvan Newman) method and detect some extra community as well. For instance, Chinesewhispers and Leiden performed well on the network dataset by identifying three communities that agree with our algorithm and multiple other communities. For the other communities, Principled clustering presented one interesting community corresponding to some parking providers helping the NHS (National Health Service) staff to get free parking during corona pandemic. With Belief and Leiden, the additional communities were about artisans and artists that paint the parking spaces and correct the lining of the spaces. Fluid and Chinesewhispers showed communities of people who lease and sell parking spaces for some price. This comparison confirmed that our choice of utilizing three detection algorithms was pretty rational and its result agree to a large extent with some state-of-the-art algorithms when applied to the same dataset where essential and critical communities have been identified.

**Table 10:** Comparison with other community detection algorithms

Algorithm	Similar communities	Other (example) community		
		Size	Hashtags	Frequent words
Principled_clustering	Marketing community Event community	11 nodes, 10 edges	#nhs, #coronavirus	nhs, social, care, free, staff
Belief	Event community	78 nodes, 91 edges	#floor, #paint	Spaces, painting, surfaces, town, street
Fluid communities	Pavement community	22 nodes, 27 edges	#passiveincome, #places, #people	Matter, reach, renters, cancel, pose
Leiden	Marketing community Event community Pavement community	8 nodes, 10 edges	#floor, #paint, #lines	Repainting, line, marking, marking, lining, green
Chinesewhispers	Marketing community Event community Pavement community	15 nodes, 19 edges	#toronto, #accessibleparking, #electricboard	Find, board, pull

### Discussions

Table 11 provides a summary of the four communities discussed in this work, highlighting their size, most frequent words, hashtags, and sentiment analysis that accompany the tweets forming each community. This sentiment analysis is added to provide more valuable insights about the polarity direction that dominates each community and to better comprehend the impact of the parking behavior.

**Table 11:** Summary of the communities with their sentiment

Community	Size	Positive sentiment	Negative sentiment	Neutral sentiment	Hashtags	Frequent words
21 core	29 nodes 372 edges	62	0	37	#parkwheels, #parking, #challenge, #contestalert, #puzzle, #findthecar, #contest	Friends, join, car, 6, ans, tagging, total
Pavement parking	35 nodes 37 edges	30	41	29	#coronavirus, #parkingnews, #pavement, #pedestrians, #accessibleparking, #parkingfail, #driving, #avementparking	Banned, pavement, pedestrians, pandemic, rule, pose, England, panel
Marketing	20 nodes 34 edges	37	42	21	#hospital, #scams, #bluebadge, #disabled, #covid19, #newport, #station	Private, spaces, charges, unfair, fight, fines, invoices, staff, permits, residents
Event	20 nodes 39 edges	31	12	56	#concert, #greenday, #mobility #hobby, #filmphotography, #light, #parking	Saturday, prebook, attending, concert, June, game, heading, forward, seeing, disappointment

In terms of community size, the 21 core community found using the k-core method is the largest one and it is characterized by overwhelmingly positive polarity opinion reflecting the users’ positive attitude that accompanied the parking experience. The second community in terms of size is the pavement parking, characterized by a rather negative sentiment. This is not surprising as the users showed dissatisfaction about the pavement usage for parking. Third community in terms of size, is the event community, which is dominated by positive opinion where users are interested in sharing, attending or engaging with concerts, exhibitions and game events. Finally Marketing community also exhibits positive polarity in overall with a focus on smart apps, health issues and transportation at wide as well as the associated costs and regulation.

The findings pointed out in this paper should not be hide some inherent limitations associated to the nature of data employed and the methodology. This is summarized into the following.

- From the data collection perspective, there is a boundary limitation in terms of number of tweets that can be collected by a single API call. Although, we systematically repeat the process several times to maximize the number of posts collected, there is still a limit in terms of how far in the past the search operation can be performed. In fact, the Rest API search in Twitter can only include a list of tweets that have been shared in the last seven days approximately. Despite this limitation, a such Twitter API search is commonly employed by researchers [70, 71].
- The use of textual similarity using Jaccard index has inherent structural limitation in the sense that it ignores other linguistic constructs that may be conveyed by the text message, which includes semantic similarity, dialogue act, entailment, negation, among others. Strictly speaking the use of Jaccard index is only motivated by the tendency to share key events by the users where the associated keywords or tokens are explicitly replicated in their tweets. This also bears some similarity with the popularity of string-based metric in sentence-to-sentence similarity measures [72]. Besides, the introduction of thresholding on Jaccard index based on network attribute (giant component) can be seen as a relaxation on the full “post” similarity according to Jaccard value.
- The use of Jaccard similarity, although popular in natural language processing applications, can be questioned. Alternatives metrics like Dice measures, cosine measures can also be valuable. Although it is difficult to identify relevant theoretical premises for choosing one specific measure over the other one, see, for instance the review paper of Gali et al. [46], we believe the impact of a given similarity measure would rather impact the choice of the threshold value but not the community result findings. A more in-depth analysis would be required to assess this observation.
- This research joins other researches in transportation, which stress on the importance of social media data to leverage the various travel user’s experiences. In this respect, Welch and Widita [73], for instance, suggest that public transport research can significantly benefit from pairing of transport big data sources with social media to infer customer satisfaction and validate hypothesizes about travel behavior.
- It should also be noted that the use of token based similarity is well-motivated in the context of our study. This is because the use of short text messages in tweets make the use of advanced semantic analysis somehow less relevant since most of the users do not elaborate on their opinions and thoughts so that stressing on common terms, like name of events, parking infrastructures and organisations seems a more cautious attitude to grasp the similarity among tweet messages.
- It should be noted that in the era of social media, many key players in the car parking industry have already active Twitter account with several followers. This includes operators working on car parking mobile apps, digital parking installation operators, construction operators and many other associated services. Therefore, it is not excluded that many of the populated hashtags are also created and populated by these operators in order to reach wider audience. On the other hand, one shall also mention the growing importance of bots in the data collection process as many Twitter IDs were mainly interested to create a buzz around the defined topic to increase the number of followers for business perspective. Although the full detail of the impact of the bots on the collected data is beyond the scope of this paper, extrapolating from previous research findings (see, for instance, European Commission report [74]), it is estimated a reasonable percentage of the Twitter ID are bots originated.
- The use of engagement assessment in our trend analysis presents some opportunity to handle the above bots or echo-chamber effect because it is highly believed that bots messages will be less subject to retweets or Likes. Therefore, one can rationally claim the findings of this global trend analysis are less obstructed by echo-chamber effects.
- The availability of the twitter dataset at various levels of mobile car parking apps can provide a rough indication to tackle the problem of technology adoption from social media perspective in line with research carried out in [75]. Although this research is part of our future agenda, there are sufficient ingredients to believe that a such approach is tenable and can be benefit both the service suppliers and policy-makers. Various other works were done at our research group concerning parking behavior analysis. A recent work [76] investigates the parking behavior in Finland using news articles mining approach. Some key differences between these two works concerns both the nature of input and the methodology. In Arhab et al. [76], the inputs were collected from News API, where long text documents were collected. This provides opportunity to apply more in-depth natural language processing techniques exploiting the semantic aspect and discourse to derive insights regarding user’s parking concerns and experience.

## Conclusion

In this paper, the parking behavior was examined based on social network analysis, utilizing a parking dataset gathered from twitter when tracking popular car parking related hashtags. A graph-based on similarity was constructed using Twitter user’s ID, taking into account the similarity of their tweet messages according to Jaccard similarity score. Several community detection algorithms; namely, Clique, k-core, and Girvan–Newman were combined with rational interpretation based approach that makes use of frequency of common keywords, hashtags as well as location of users in order to generate interpretale (sub) communities. This expects us to provide insights into identifying individuals’ parking behavior and factors influencing their parking search decisions. In parallel, a global trend analysis that investigates different parking types and the most engaging discussion in terms of presence of retweets or Like has been carried out. This analysis makes use of sentiment polarity and dominant discussion trends according to KeyBERT model. Some of the findings confirmed some already established results in terms of influence of discount, free parking availability on the parking search decisions. Factors related to events occurring in city have also found to influence the online pre-booking with mobile apps rather than traditional systems.

Furthermore, surprisingly, individuals’ parking skills were found to constitute a big topic as well as malicious behavior such as pavement parking. In addition, marketing behavior about parking revealed to have an important impact as well. Another community revealed that the big influencers in the parking domain are most likely to be parking providers and marketing specialists. Finally, the corona-virus pandemic has affected the traffic and the functioning of parking systems, especially near hospitals, where there was much solidarity between people to help care workers access parking lots. Besides, the developed approach for mining hastags through semi-automated process of community detection can be extrapolated to several other domain applications, with a potential high societal impact. It also joins the recently promoted concept of Explainable AI, where explanability and interpretability are seen as critical for further AI application development. Therefore, areas of future research include the hybridization of some known explainable AI approaches in model approximations, visualization and community detection in social networks.

## Data Availability

Both data and code are made available from Github account of the first author.

## Appendix

*Network Features* Next, the main network features/attributes of the graph (either the whole graph or subgraph corresponding to individual community detected using e.g., Clique, K-core or Girvan–Newman) are computed.

Average Path Length measures the mass transport on the network and is defined as the average number of steps along the shortest paths for all possible pairs of network nodes [77]. More formally, this boils down to the following expression:

$$a_G = \sum _{s,t \epsilon V}\frac{d(s,t)}{n(n-1)}$$

*V* is the set of all nodes of the graph (sub-graph). *s* and *t* are two nodes of the graph

Average Degree Centrality is calculated as the arithmetic average of individual node degree centralities. The degree centrality of a given node is determined by counting the number of edges connected to this specific node [78]:

$$C_{d_{avg}} = \frac{1}{n} \sum _{i=1}^n C_d(v_i)$$

$C_d(v_i) = \frac{d_i}{n-1}$, where $d_i$ corresponds to the degree of node $v_i$, and *n* is total number of nodes in the graph (sub-graph).

Average In-betweenness Centrality captures how much a given node, say u, is in-between others. This is measured with the number of shortest paths (between any couple of nodes in the graphs) that passes through the target node u. The average in-betweenness centrality provides the average across all nodes of the graph [77]. Formally, this boils down to:

$$C_{b_{avg}} = \frac{1}{n} \sum _{i=1}^n C_b(v_i)$$

$C_b(v_i) = \sum _{s\ne t\ne v_i} \frac{\sigma _{st}(v_i)}{\sigma _{st}}$. $\sigma _{st}(v_i)$ are the counts of the shortest paths of *s* and *t* passing through $v_i$. $\sigma _{st}$, and *n* total number of nodes.

Clustering coefficient provides an estimate of how much a node tends to form triangles with other nodes in the graph. This yields a measure of the density of the connection; that is, higher the clustering coefficient, high the connection density [79]. More formally, it can also be expressed in terms of local clustering coefficient for individual nodes:

$$C_{{}} = \frac{1}{n} \sum _{i=1}^n C_{loc}(v_i)$$

where $C_{loc}(v_i) = \frac{2T(v_i)}{d_i(d_i-1)}$. $T(v_i)$ indicates the number of triangles connected to node $v_i$. $d_i$ is the degree of node $v_i$

Diameter to get the diameter, first shortest paths between node pairs is calculated, after that, the average is taken, then the diameter is defined [80] by

$$diam_G = max_{(s,t)\epsilon V}$$

where *V* be the set of nodes in the social network, and *d*(*s*, *t*) be the shortest path between nodes *s* and *t*.

## Footnotes

1 We mean by “parking system” the technology employed to monitor the availability of parking lots, opening/closure of parking gate and communication with user, if any.

2 https://pypi.org/project/GetOldTweets3/.

3 We used the default of English stop-word list in NLTK library after an initial manual scrutinization to avoid discarding words, which can impact the parking understanding reports.

4 It should be noted that our implementation of Jaccard measure is at token (word) level, not at character level as in the original NLTK implementation.

5 https://networkx.github.io/.

6 http://www.parkingspacerentals.co.uk/.
